# Health and Economic Benefits of Complying With the World Health Organization Air Quality Guidelines for Particulate Matter in Nine Major Latin American Cities

**DOI:** 10.3389/ijph.2024.1606909

**Published:** 2024-05-30

**Authors:** Lina Madaniyazi, Jefferson Alpízar, Luis Abdón Cifuentes, Horacio Riojas-Rodríguez, Magali Hurtado Díaz, Micheline de Sousa Zanotti Stagliorio Coelho, Rosana Abrutzky, Samuel Osorio, Gabriel Carrasco Escobar, Nicolás Valdés Ortega, Valentina Colistro, Dominic Roye, Aurelio Tobías

**Affiliations:** ^1^ School of Tropical Medicine and Global Health, Nagasaki University, Nagasaki, Japan; ^2^ Departamento de Ingeniería, Pontificia Universidad Católica de Chile, Santiago, Chile; ^3^ National Institute of Public Health (Mexico), Cuernavaca, Mexico; ^4^ Urban Health Laboratory University of São Paulo, Faculty of Medicine, São Paulo, Rio Grande do Sul, Brazil; ^5^ Universidad de Buenos Aires, Facultad de Ciencias Sociales, Instituto de Investigaciones Gino Germani, Buenos Aires, Argentina; ^6^ Health Innovation Laboratory, Institute of Tropical Medicine “Alexander von Humboldt”, Universidad Peruana Cayetano Heredia, Lima, Peru; ^7^ Department of Quantitative Methods, School of Medicine, University of the Republic, Montevideo, Uruguay; ^8^ CIBER de Epidemiología y Salud Pública (CIBERESP), Madrid, Spain; ^9^ Institute of Environmental Assessment and Water Research, Spanish National Research Council (CSIC), Barcelona, Spain

**Keywords:** air pollution, particulate matter, mortality, Latin America, air quality regulation, economic benefits

## Abstract

**Objectives:**

This study aims to estimate the short-term preventable mortality and associated economic costs of complying with the World Health Organization (WHO) air quality guidelines (AQGs) limit values for PM_10_ and PM_2.5_ in nine major Latin American cities.

**Methods:**

We estimated city-specific PM-mortality associations using time-series regression models and calculated the attributable mortality fraction. Next, we used the value of statistical life to calculate the economic benefits of complying with the WHO AQGs limit values.

**Results:**

In most cities, PM concentrations exceeded the WHO AQGs limit values more than 90% of the days. PM_10_ was found to be associated with an average excess mortality of 1.88% with concentrations above WHO AQGs limit values, while for PM_2.5_ it was 1.05%. The associated annual economic costs varied widely, between US$ 19.5 million to 3,386.9 million for PM_10_, and US$ 196.3 million to 2,209.6 million for PM_2.5_.

**Conclusion:**

Our findings suggest that there is an urgent need for policymakers to develop interventions to achieve sustainable air quality improvements in Latin America. Complying with the WHO AQGs limit values for PM_10_ and PM_2.5_ in Latin American cities would substantially benefits for urban populations.

## Introduction

In recent decades, Latin American urban centers have witnessed rapid urbanization and industrialization, leading to a surge in air pollution levels [1]. Among the various pollutants, particulate matter (PM) has emerged as a critical public health concern, given its harmful impact on respiratory and cardiovascular systems [2–4]. The World Health Organization (WHO) has established air quality guidelines (AQGs) for particulate matter (PM), aiming to safeguard human health and well-being [5]. In particular, the WHO AQGs recently updated the annual limit values for particulate matter with an aerodynamic diameter of 10 μm or less (PM_10_) to 15 $\mu g/m^{3}$ , and for particulate matter with an aerodynamic diameter of 2.5 μm or less (PM_2.5_) to 5 $\mu g/m^{3}$ . However, compliance with these stringent standards remains a significant challenge for major Latin American cities, where factors such as population density, traffic congestion, industrial emissions, and limited resources for environmental management converge.

Understanding the multifaceted implications of non-compliance with WHO AQGs is essential for designing effective mitigation strategies [6]. One crucial aspect of this assessment is the short-term preventable mortality associated with elevated PM levels, especially in Latin American urban centers. While the total burden attributed to long-term exposure far exceeds that of short-term exposure, the immediacy of the latter presents a distinct contrast. Unlike the gradual realization of benefits associated with improved air quality over months and years due to long-term exposure, short-term effects can be mitigated “immediately.” Consequently, policies targeting the reduction of daily concentrations will promptly yield benefits in terms of short-term effects, whereas the broader advantages of enhanced air quality will materialize only over an extended and less precisely defined period. Moreover, the economic ramifications of failing to comply with WHO AQGs demand rigorous investigation. The cost burden extends across various sectors, including healthcare expenditures, loss of labor productivity, and diminished quality of life [7]. By quantifying the economic burden of air pollution, policymakers can make informed decisions regarding resource allocation and prioritize interventions to achieve sustainable air quality improvements.

This study aims to present a comprehensive analysis of short-term preventable mortality and associated economic costs of complying with the WHO AQGs for PM_10_ and PM_2.5_ in nine major Latin American cities. These health and economic consequences of PM pollution offer a foundation for evidence-based policy formulation to enhance air quality and preserve the wellbeing of urban populations in Latin America.

## Methods

### Data Collection

**FIGURE 1:**
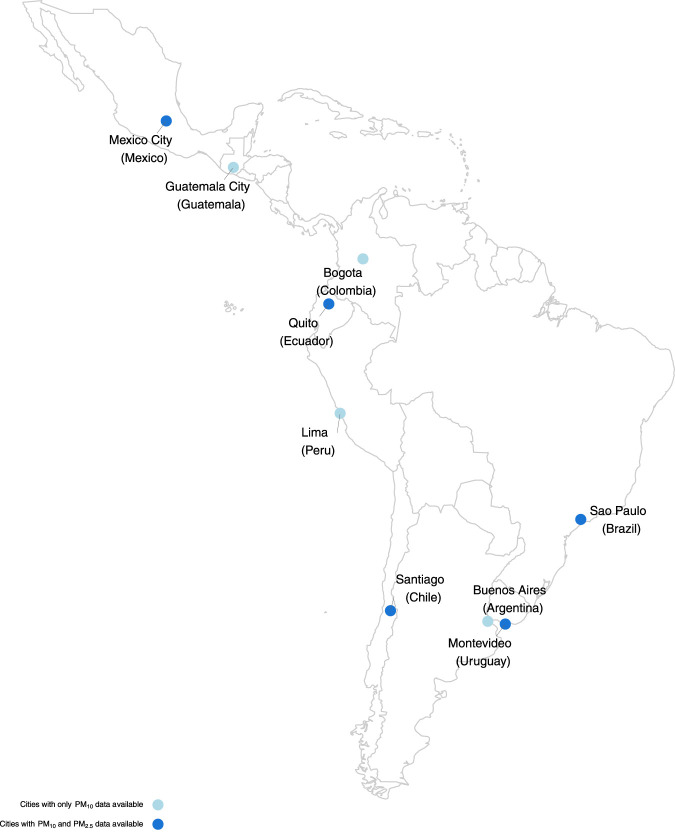
Geographical location of the cities in the study (Latin America, 2009–2018). Note: PM_10_: Particulate matter with an aerodynamic diameter of 10 μm or less. PM_2.5_: Particulate matter with an aerodynamic diameter of 2.5 μm or less.

We collected daily time series data on environment and health from nine capital cities or the most populated cities in Central and South American countries (Figure 1), namely, Bogota in Colombia, Buenos Aires in Argentina, Guatemala City in Guatemala, Lima in Peru, Mexico City in Mexico, Montevideo in Uruguay, Quito in Ecuador, Santiago in Chile, and Sao Paulo in Brazil. The dataset covers an overlapping period from 2009 to 2018. Mortality data were obtained from local authorities within each country, represented by daily counts of deaths due to non-external causes (International Classification of Diseases, 9th revision (ICD-9) codes 0 to 799 and ICD-10 codes A0 to R99). In cases where non-external mortality data were unavailable, we collected daily counts of deaths from all causes. We obtained daily concentrations of PM_10_ in nine cities, and on PM_2.5_ from five of these cities. Data on both pollutants were available in Mexico City, Montevideo, Quito, Santiago, and Sao Paulo. We also collected data on the daily mean temperature for each city. Data on PM and temperature were all collected from local monitoring stations and networks in each city.

### Statistical Analysis

The analysis included three steps. First, we estimated the PM-mortality association, and then derived the health impact. Finally, we calculated the economic benefits of complying with WHO AQGs limit values for PM concentrations. The analysis was conducted for PM_10_ and PM_2.5_ separately using R (version 4.3.1; R Development Core Team).

### PM-Mortality Association

We performed city-specific time-series analyses using generalized linear models with quasi-Poisson family [8]. We developed the model based on a previous study [9]. The regression model included a natural cubic spline function with 7 degrees of freedom (*df*) per year to control for the long-term trends and seasonality and an indicator for the day of the week to account for within-week variation. We used a natural cubic spline function with 6 *df* for the 4-day moving average of daily mean temperature to account for its confounding effect on PM-mortality associations. We assumed a linear exposure-response association of mortality with PM. To identify the optimal lag days (i.e., the number of days the effect of PM could persist), we used distributed linear models with a natural cubic spline with 3 *df* for the lag-response association for the same day (lag 0) to four days after the exposure (lag 4). Then we pooled the city-specific estimates for the association by using a random-effects meta-analysis by considering city as a random effect. We reported relative risk (RR) of mortality, and the related 95% confidence interval (95%CI), for a 10 $\mu g/m^{3}$ increase of PM_10_ and PM_2.5_.

### Attributable Mortality

Although we focus on the short-term association between PM and mortality, we utilized the WHO AQGs annual limit value rather than the daily limit value to estimate the attributable mortality. The 2021 WHO AQGs were specifically determined to ensure compliance with the more crucial long-term limit values and the regulations governing daily levels [5]. Essentially, areas that meet the annual AQG limit value are likely also to meet the requirement of not surpassing the daily limit values more than three times a year and *vice versa* [10]. Therefore, any impact assessment must recognize this consistency: if every day of the year, on average, adheres to the long-term limit value, both the long-term and the short-term AQG values will be met; conversely, if every day was, on average, aligns with the short-term daily mean limit values, the AQG annual mean limits would be significantly violated. Hence, the only appropriate reference values to derive the burden for “non-compliance” with WHO AQGs are the long-term mean limit values (5 and 15 μg/m^3^, respectively for PM_2.5_ and PM_10_), not the short-term limit values [10].

We calculated the attributable mortality associated with the short-term exposure to PM_10_ and PM_2.5_ in each city for days above the WHO AQGs annual limit values as $\left(1-\exp\;\left({-\beta }_{i}\cdot |{\left(x_{it}-c\right)}^{+}\right)\right)\times d_{it}$ . Here, $\beta_{i}$ is the log-RR for a unit increase in PM concentration in city i , $x_{it}$ represents the daily PM concentration in city i on day t , c is the WHO AQGs limit value ( $c=15\;\mu /m^{3}$ for PM_10_, and $c=5\;\mu /m^{3}$ for PM_2.5_), and $d_{it}$ is the daily deaths in city i on day t . Finally, we computed the mortality fraction (%) by summing of the city-specific daily attributable deaths and dividing by the total mortality in each city, allowing for the comparison across cities, jointly with the 95% empirical CIs (eCIs) [11].

### Economic Cost

We employed the concept of the value of a statistical life (VSL) to calculate the economic benefits associated with the reduction of PM_10_ and PM_2.5_ levels in each city. The VSL serves as a widely used measure in cost-benefit analyses, assessing the health cost related to both environment and healthcare programs that influence social wellbeing, such as the health cost of deaths attributable to PM pollution. Essentially, VSL represents an individual’s willingness to pay to reduce a unit of mortality risk [12]. To quantify the economic benefits of PM reduction, we multiplied the VSL by the number of attributable deaths in each city and calculated the average cost per year.

Ideally, the VSL obtained from the local empirical studies should be used for the calculation. However, such information was not available for the current study. Therefore, to ensure comparability between countries, we relied on international income-adjusted estimates of the country specific VSL [12] as a proxy for the economic cost in each city. The city-specific estimates were reported as annual average number of excess deaths and annual average economic benefits, allowing for a proper comparison between cities with different lengths of study period.

## Results

The analysis included 2,582,439 deaths across nine major cities in Latin America, with the period ranging from three to ten years. Table 1 shows the descriptive summary of PM_10_ and PM_2.5_ concentrations, average temperature, and daily mortality in each city. On average, the annual mean concentrations of PM_10_ ranged from 27.4 $\mu g/m^{3}$ in Montevideo to 77.9 $\mu g/m^{3}$ in Lima, while PM_2.5_ concentrations ranged from 8.6 $\mu g/m^{3}$ in Montevideo to 27.4 $\mu g/m^{3}$ in Santiago. Across the cities and study periods, 97.4% and 97.0% of days showed concentrations of PM_10_ and PM_2.5_ above the WHO AQGs daily limit values of 15 $\mu g/m^{3}$ and 5 $\mu g/m^{3}$ , respectively. Majority of the days (>90%) in most cities recorded concentrations of PM higher than the WHO AQGs limit values, except for PM_2.5_ in Montevideo (Supplementary Figure S1).

**TABLE 1:** Descriptive summary by city (Latin America, 2009–2018).

City (country)	Study period	Deaths	Mean (standard Deviation)			Percentage of days PM_10_ > 15 $\mu g/m^{3}$ (%)	Percentage of days PM_2.5_ > 5 $\mu g/m^{3}$ (%)
			Temperature ( ℃ )	PM_10_ ( $\mu g/m^{3}$ )	PM_2.5_ ( $\mu g/m^{3}$ )		
Bogota (Colombia)	2009–2013	142,151	14.1 (0.9)	53.2 (15.8)	-	100	-
Buenos Aires (Argentina)	2009–2018	399,592	18.3 (5.8)	29.6 (15.3)	-	94.3	-
Guatemala City (Guatemala)	2010–2015	48,170	19.3 (1.5)	48.0 (26.3)	-	98.2	-
Lima (Peru)	2010–2014	183,105	19.2 (2.4)	77.9 (26.1)	-	100	-
Mexico City (Mexico)	2009–2014	610,387	16.5 (2.5)	51.2 (20.4)	24.0 (9.9)	99.3	100
Montevideo (Uruguay)	2014–2016	92,252	18.6 (5.4)	27.4 (11.2)	8.6 (8.0)	91.4	67.1
Quito (Ecuador)	2014–2018	44,533	15.5 (1.1)	47.7 (17.3)	16.7 (5.3)	98.7	100
Santiago (Chile)	2009–2018	380,102	15.0 (5.1)	69.8 (32.1)	27.4 (15.5)	99.0	99.6
Sao Paulo (Brazil)	2010–2018	682,147	21.5 (3.5)	36.3 (18.0)	20.8 (10.9)	95.2	99.6

PM_10_: Particulate matter with an aerodynamic diameter of 10 μm or less.

PM_2.5_: Particulate matter with an aerodynamic diameter of 2.5 μm or less.

The lag-response association for most of the cities suggested a consistent delayed effect of PM on the current day (lag 0) and 1 day before (lag 1) (Supplementary Figures S2–S3). Therefore, we fitted a linear exposure-response association of mortality with the 2-day moving average of daily concentration of PM (lag 0–1 day) and observed a positive association between PM and mortality in all cities (Supplementary Figures S4–S5).

**FIGURE 2:**
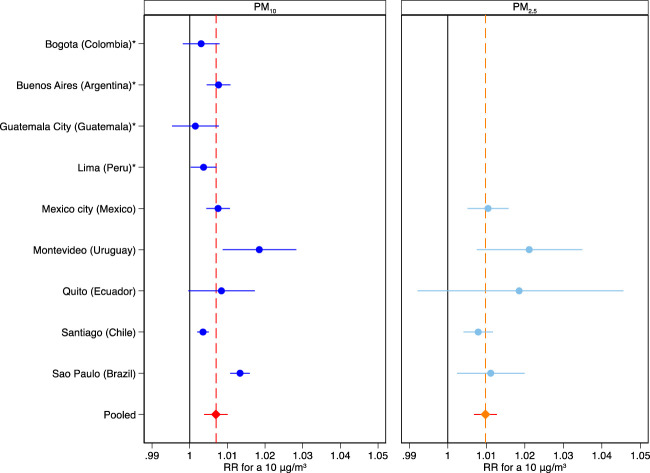
Pooled and city-specific short-term association of mortality with 2-day moving average concentration of particulate matter, as relative risk (RR, and 95% confidence interval) for a 10 $\mu g/m^{3}$ increase (Latin America, 2009–2018). Note: PM_10_: Particulate matter with an aerodynamic diameter of 10 μm or less. PM_2.5_: Particulate matter with an aerodynamic diameter of 2.5 μm or less. *Cities with only PM10 data available.

The pooled estimate showed that an increase of 10 $\mu g/m^{3}$ in PM_10_ was associated with a RR of 1.007 (95%CI= [1.004, 1.010]), while PM_2.5_ was associated with a RR of 1.010 (95%CI= [1.007%, 1.013%]) (Figure 2; Supplementary Table S1). The city-specific RRs varied among cities, ranging from 1.001 (95%CI= [0.995, 1.008]) in Guatemala City to 1.018 (95%CI= [1.009, 1.028]) in Montevideo for PM_10_ and from 1.008 (95%CI= [1.004, 1.012]) in Santiago to 1.021 (95%CI= [1.008, 1.035]) in Montevideo for PM_2.5_.

**FIGURE 3:**
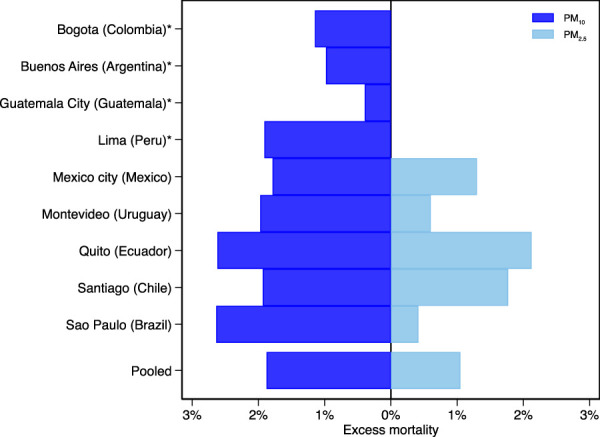
Pooled and city-specific excess mortality associated with particulate matter concentrations above the World Health Organization air quality guidelines limit (Latin America, 2009–2018). Note: The World Health Organization air quality guidelines limit values are 15 $\mu g/m^{3}$ for particulate matter with an aerodynamic diameter of 10 μm or less (PM_10_) and 5 $\mu g/m^{3}$ for particulate matter with an aerodynamic diameter of 2.5 μm or less (PM_2.5_). *Cities with only PM10 data available.

Overall, PM_10_ and PM_2.5_ were found to be associated with an excess mortality of 1.88% (95% eCI = [1.02, 2.76]) and 1.05% (95% eCI = [0.42, 1.70]) with levels above the WHO AQGs limit values, respectively (Figure 3; Supplementary Table S2). For PM_10_, city-specific excess mortality ranged from 0.39% (95%eCI = [−1.30, 2.13]) in Guatemala to 2.62% (95%eCI = [−0.17, 5.46]) in Quito, while for PM_2.5_, city-specific excess mortality ranged from 0.42% (95%eCI = [0.08, 0.76]) in Sao Paulo to 2.13% (95%eCI = [−0.99, 5.29]) in Quito. It should be noted that the estimates in Bogota, Guatemala City, and Quito exhibit some uncertainties.

Table 2 shows the city-specific annual average estimates on excess deaths and economic cost associated with PM concentrations above the WHO AQGs limit values. The annual average economic costs of PM_10_ varied widely from US $19.5 million (95%eCI = [−64.4, 105.4]) in Guatemala City to US$ 3,386.9 million (95%eCI = [2,728.7, 4,076.2]) in Sao Paulo. Similarly, for PM_2.5_, costs ranged from US$ 196.3 million (95%eCI = [−91.7, 488.8]) in Quito to US$ 2,209.6 million (95%eCI = [1,072.3, 3,396.1]) in Mexico City. Notably, Mexico City, Santiago, and Sao Paulo showed the heaviest economic burden, exceeding both PM_10_ and PM_2.5_ WHO AGQs limit values.

**TABLE 2:** City-specific average annual excess deaths and annual economic cost associated with particulate matter concentrations above the World Health Organization air quality guidelines limit (Latin America, 2009–2018).

		PM_10_		PM_2.5_	
City (country)	VSL (US$ million)	Excess deaths (n, (95%eCI))	Economic cost (US$ million, (95%eCI))	Excess deaths (n, (95%eCI))	Economic cost (US$ million, (95%eCI))
Bogota (Colombia)^a^	1.228	326 (−210; 880)	400.5 (−257.5; 1,080.8)		
Buenos Aires (Argentina)^a^	2.144	391 (228; 563)	839.4 (488.4; 1,206.9)		
Guatemala City (Guatemala)^a^	0.618	32 (−105; 171)	19.5 (−64.6; 105.4)		
Lima (Peru)^a^	1.055	699 (33; 1,386)	737.7 (34.8; 1,461.7)		
Mexico City (Mexico)	1.671	1,818 (1,056; 2,610)	3,037.3 (1,765.1; 4,361.8)	1,322 (642; 2032)	2,209.6 (1,072.3; 3,396.1)
Montevideo (Uruguay)	2.705	607 (285; 940)	1,641.2 (770.6; 2,541.4)	185 (65; 309)	501.6 (175.9; 836.7)
Quito (Ecuador)	1.037	233 (−16; 487)	241.9 (−16.1; 504.7)	189 (−88; 471)	196.3 (−91.7; 488.8)
Santiago (Chile)	2.426	735 (407; 1,078)	1,782.6 (986.9; 2,614.1)	673 (345; 1,016)	1,633.6 (838.0; 2,463.8)
Sao Paulo (Brazil)	1.695	1,998 (1,610; 2,405)	3,386.9 (2,728.7; 4,076.2)	316 (64; 577)	535.5 (108.7; 977.4)

Note: The WHO AQGs limit values are 15 $\mu g/m^{3}$ for particulate matter with an aerodynamic diameter of 10 μm or less (PM_10_) and 5 $\mu g/m^{3}$ for particulate matter with an aerodynamic diameter of 2.5 μm or less (PM_2.5_).

^a^ Cities with only PM10 data available.

## Discussion

This study analyzes data on PM and daily mortality in nine major cities in Latin America, providing evidence of the health and economic impact of daily PM concentrations above the WHO AQGs recently updated limit values for PM_10_ and PM_2.5_.

In the analysis, we observed a risk increase of 0.7% in all-cause mortality per 10 $\mu g/m^{3}$ increase in PM_10_ and 1% for PM_2.5_. These risk estimates are similar to those reported in previous studies in Latin American countries. The ESCALA study reported a mortality risk of 0.77% for PM_10_ [13], while a systematic review estimated a pooled mortality risk of 1% for PM_2.5_ [2]. However, the short-term effects of PM in Latin America are somewhat larger than those described in studies at the global scale. For example, Liu et al. [9] reported risk increases of 0.44% and 0.68% for PM_10_ and PM_2.5_, respectively, in a study from 652 cities in 24 countries. Similarly, a WHO systematic review reported risk increases of 0.41% for PM_10_ and 0.65% for PM_2.5_ [3]. However, the city-specific risk estimates showed geographical variability, ranging between 0.1% and 1.8% for a 10 $\mu g/m^{3}$ increase of PM_10_, and 0.8% to 2.1% for PM_2.5_. These may be related to city-specific demographics, such as variations in age distribution, socio-economic development, PM sources, and climate conditions. However, further studies are warranted to investigate these variations.

Nevertheless, our study offers a new perspective on the impact of short-term exposure to PM pollution in Latin America by estimating the health burden and its economic consequences at the city level, an investigation lacking in previous studies. In most cities studied, PM concentration exceeded WHO AQGs limit values on over 90% of days. In several cities, daily average PM_10_ and PM_2.5_ concentration consistently surpassed WHO AQG limit values.

In addition, PM_10_ and PM_2.5_ are associated with a short-term excess mortality of 0.39% and 0.43%, respectively, with levels above the current WHO AQGs limit values. This implies an estimated annual economic cost, which varies widely between US $19.5 to 3386.9 million for PM_10_, and US $196 to 2209.6 million for PM_2.5_. However, comparisons with previous studies are not straightforward since previous studies estimating deaths attributable to ambient PM mainly focused on the long-term effects, which are much larger than our estimation of the short-term effects. A regional multi-city study including 366 Latin American cities revealed that 58% of the population lived in the areas where annual PM_2.5_ concentrations surpassed the 2005 WHO AQG of 10 $\mu g/m^{3}$ [14]. Moreover, the State of Global Air estimated the number of deaths attributable to long-term exposure to PM_2.5_ in the Latin American countries considered in our study ranged between 733 in Uruguay to 43,600 in Brazil [15]. Similarly, different methods have been used to estimate economic impact. For example, Trejo-González et al. [16] estimated that an average reduction of 10 $\mu g/m^{3}$ in the annual PM_2.5_ in fifteen cities in Mexico during 2015 would have prevented 14,666 deaths and 150,771 potential years of life lost in 2015, with estimated costs of US $64,164 and $5,434 million, respectively. A recent study reported US$ 148.3 billion could be attributed to productivity lost due to PM_2.5_ above the 2021 WHO AQGs in Brazil between 2000 and 2019 [17]. Moreover, Bell et al. [18] estimated the economic benefits of PM pollution reduction under two emission scenarios in Mexico City, Santiago, and Sao Paulo using willingness-to-pay and cost-of-illness from 2000 to 2020 for two emission scenarios based on current emissions patterns and regulatory trends and a control policy aimed at lowering air pollution, which was roughly US $21 to $165 billion.

Despite methodological disparities, our findings, coupled with previous studies, underline the substantial health burden and the associated economic cost posed by air pollution in Latin American urban centers, underscoring the significant benefits of lowering the PM concentrations to the current WHO AQGs limit values [6]. However, the urbanization process continues to increase in Latin American countries [1]. The primary factors leading to the deteriorating air quality in the region are the vehicle fleet, industrial sources, and biomass burning [14]. The combustion of solid fuels for cooking or heating within households adds to the overall air pollution in urban areas of certain countries, particularly where a significant portion of the population relies on solid fuels as their primary energy source [1]. These may lead governments to consider that updating their national ambient air quality standards to achieve the newly updated WHO AGQs limit values may not be feasible in their local context at the short term. Here, the interim targets proposed by WHO may be useful steps toward a progressive reduction of PM concentrations [5].

It is necessary for the countries in the region to update the regulatory framework for air quality, especially for PM, to protect public health and the environment. This should include sustainable solutions for public transportation and mobility, as well as the promotion of sustainable clean energy [1]. In this context, countries like Mexico, Colombia, and Brazil have approached their air quality standards to the WHO guidelines. However, the regulatory framework should also include the emission standards, which have a considerable delay for the countries in the region, as well as regulations related to the specifications of the fuels used.

Several limitations should be acknowledged. Single-pollutant models were fitted because data for gaseous pollutants (i.e., nitrogen and sulfur dioxides) were unavailable. In a global study, Liu et al. [9] found that the magnitude of the PM_10_ and PM_2.5_ associations with all-cause mortality, although they remained statistically significant, decreased after adjusting for gaseous pollutants. Moreover, we used time-series analysis to derive the concentration-response associations of short-term exposure to PM. It is important to note that this approach may lead to an underestimation of the potential health and economic impact associated with reducing PM concentrations. Time-series studies capture only cases in which death has been triggered by air pollution exposure incurred shortly before death [19]. For instance, we observed a risk of 1% in all-cause mortality per 10 $\mu g/m^{3}$ increase in PM_2.5_ which is notably smaller compared to the 9% estimated for the long-term exposure [20]. Furthermore, the short-term effect of PM on all-cause mortality is merely the tip of the iceberg, ignoring numerous other acute health outcomes and diseases, such as myocardial infarctions and cardiorespiratory acute hospitalizations, that are also linked to PM exposure. Therefore, we recommend future studies include other acute health outcomes and extend the current analysis to assess the long-term effects of air quality improvement related to PM in Latin America.

In conclusion, the findings reported in this study show noteworthy evidence that there is an urgent need for policymakers to develop more ambitious policies aimed at achieving sustainable air quality improvements in Latin America. Complying with the WHO AQGs daily limit values for PM_10_ and PM_2.5_ would provide substantial benefits for the urban populations in Latin American cities.
