# Supplementary material for: Health and Economic Benefits of Complying With the World Health Organization Air Quality Guidelines for Particulate Matter in Nine Major Latin American Cities
Source: Int J Public Health. 2024 May 30;69:1606909. doi: 10.3389/ijph.2024.1606909 (PMC11176932; doi:10.3389/ijph.2024.1606909)
Supplement: Supplementary file 1 [file DataSheet1.docx]

**Supplementary Material**

**Health and economic benefits of complying with the World Health Organization Air Quality Guidelines limit values for particulate matter in nine major Latin American cities**

**Table S1.** Pooled and city-specific short-term association of mortality with 2-day moving average concentration of PM_10_ and PM_2.5_, as relative risk (RR, and 95% confidence interval) for a 10 $\mu g/m^{3}$ increase.

|  | PM_10_ | |  | PM_2.5_ | |
| --- | --- | --- | --- | --- | --- |
| City (Country) | RR | (95%CI) |  | RR | (95%CI) |
| Bogota (Colombia) | 1.003 | (0.998, 1.008) |  |  |  |
| Buenos Aires (Argentina) | 1.008 | (1.004, 1.011) |  |  |  |
| Guatemala City (Guatemala) | 1.001 | (0.995, 1.008) |  |  |  |
| Lima (Peru) | 1.004 | (1.000, 1.007) |  |  |  |
| Mexico City (Mexico) | 1.008 | (1.004, 1.011) |  | 1.010 | (1.005, 1.016) |
| Montevideo (Uruguay) | 1.018 | (1.009, 1.028) |  | 1.021 | (1.008, 1.035) |
| Quito (Ecuador) | 1.008 | (1.000, 1.017) |  | 1.019 | (0.992, 1.046) |
| Santiago (Chile) | 1.004 | (1.002, 1.005) |  | 1.008 | (1.004, 1.012) |
| Sao Paulo (Brazil) | 1.013 | (1.011, 1.016) |  | 1.011 | (1.002, 1.020) |
| Pooled | 1.007 | (1.004, 1.010) |  | 1.010 | (1.007, 1.013) |

**Table S2**. Pooled and city-specific excess mortality (AF%) associated with PM concentrations above the WHO AQGs limit values of 15 $\mu g/m^{3}$ for PM_10_ and 5 $\mu g/m^{3}$ for PM_2.5_

|  | PM_10_ | |  | PM_2.5_ | |
| --- | --- | --- | --- | --- | --- |
| City (Country) | AF% | (95%eCI) |  | AF% | (95%eCI) |
| Bogota (Colombia) | 1.15 | (-0.74, 3.10) |  |  |  |
| Buenos Aires (Argentina) | 0.98 | (0.57, 1.41) |  |  |  |
| Guatemala City (Guatemala) | 0.39 | (-1.30, 2.13) |  |  |  |
| Lima (Peru) | 1.91 | (0.09, 3.78) |  |  |  |
| Mexico City (Mexico) | 1.79 | (1.04, 2.57) |  | 1.30 | (0.63, 2.00) |
| Montevideo (Uruguay) | 1.97 | (0.93, 3.06) |  | 0.60 | (0.21, 1.01) |
| Quito (Ecuador) | 2.62 | (-0.17, 5.46) |  | 2.13 | (-0.99, 5.29) |
| Santiago (Chile) | 1.93 | (1.07, 2.83) |  | 1.77 | (0.91, 2.67) |
| Sao Paulo (Brazil) | 2.64 | (2.12, 3.17) |  | 0.42 | (0.08, 0.76) |
| Pooled | 1.88 | (1.02, 2.76) |  | 1.05 | (0.42, 1.70) |

**Figure S1**. City-specific distribution of the PM_10_ and PM_2.5_ concentrations (in $\mu g/m^{3}$).

**Figure S2.** Lag-response association as relative risk of mortality for a 10 $\mu g/m^{3}$ increase in PM_10_ for the same day (lag 0) to four days after the exposure (lag 4).

**Figure S3.** Lag-response association as relative risk of mortality for a 10 $\mu g/m^{3}$ increase in PM_2.5_ for the same day (lag 0) to four days after the exposure (lag 4).

**Figure S4.** City-specific exposure-response associations for 2-day moving average concentration of PM_10_ centered at the WHO AQGs limit value of 15 $\mu g/m^{3}$.

**Figure S5.** City-specific exposure-response associations for 2-day moving average concentration of PM_2.5_ centered at the dashed vertical line denotes WHO AQGs limit value of 5 $\mu g/m^{3}$.
